# Recent Progress in the Removal of Legacy and Emerging Organic Contaminants from Wastewater Using Metal–Organic Frameworks: An Overview on Adsorption and Catalysis Processes

**DOI:** 10.3390/ma15113850

**Published:** 2022-05-27

**Authors:** Silviu-Laurentiu Badea, Violeta-Carolina Niculescu

**Affiliations:** National Research and Development Institute for Cryogenic and Isotopic Technologies—ICSI Ramnicu Valcea, 4th Uzinei Street, 240050 Ramnicu Valcea, Romania; silviu.badea@icsi.ro

**Keywords:** adsorption, catalysis, MOF, photocatalysis, wastewater

## Abstract

Water covers about 70% of the Earth’s surface, but the amount of freshwater available for human use is only 2.5% and, although it is continuously replenished via the water cycle, freshwater is a finite and limited resource. The Earth’s water is affected by pollution and while water quality is an issue of global concern, the specific regulations on contaminants of emerging concern (CECs) are limited. In order to achieve the goals set by EU regulations, the treatment of wastewater is a scientifically and technologically challenging issue. Metal–organic frameworks (MOFs) are promising materials used for the removal of priority and emerging contaminants from wastewater, since they can mitigate those contaminants via both adsorption as well as catalysis processes. MOFs can offer selective adsorption of CECs by various adsorption mechanisms. The catalytic removal of priority and emerging organic contaminants from wastewater using MOFs implies Fenton, electro-Fenton, and photo-Fenton processes. Overall, MOFs can be considered as promising materials for the elimination of priority and emerging organic contaminants from various wastewater types, but the involved processes must be studied in detail for a larger number of compounds.

## 1. Introduction

The occurrence of micro-pollutants in the environment is considered an environmental emergency across the globe. Contaminants of emerging concern (CECs) is a term used by water quality professionals to describe pollutants that have been detected in water bodies, may cause ecological or human health impacts, and typically are not regulated under current environmental laws. Sources of these pollutants include agriculture, urban runoff, and ordinary household products and pharmaceuticals that are disposed to wastewater treatment plants (WWTPs) and subsequently discharged to surface waters. Consistently, various studies have shown the presence of pollutants in influents and effluents of WWTPs and in receiving waters due to their incomplete removal by wastewater treatment processes [1]. Because of these, many emerging compounds are also being added to the EU Water Framework Directive (2000/60/EC) (WFD) priority substance list and an emerging watch list, increasing the significance of monitoring these chemicals. Examples of typical priority organic pollutants include organochlorine pesticides such as hexachlorocyclohexanes (HCHs) and legacy brominated flame retardants such as brominated biphenyl ethers (PBDEs). Among thousands of emerging compounds, examples include a variety of pharmaceuticals [2] and novel brominated flame retardants, as well as a variety of personal care products such as triclosan and triclocarban.

In order to achieve targets set by the EU WFD and by EU Decision 2015/495 [3] (which established a watch list of contaminants of emerging concern), the treatment of wastewater is creating scientific and technological challenges. Traditional wastewater management methods using microorganisms (biodegradation) and/or physico-chemical processes (flocculation, chlorination, and ozonation), subsequently followed by filtration- and adsorption-based separations, are able to treat the majority of anthropogenically polluted water sources. Among these processes, wastewater treatment enhanced with activated carbon treatment appears to be one of the most promising mitigation methods for CECs [4].

Nevertheless, no single method described above is efficient enough to produce water with legally and practically acceptable levels of refractory toxic chemicals (e.g., phenols, dyes, pesticides, organic solvents, pharmaceuticals, and domestic chemicals). Advanced oxidation processes (AOPs) [5,6] constitute chemical treatment procedures designed to remove organic (and sometimes inorganic) substances in water and wastewater by oxidation through reactions with hydroxyl radicals (OH). Compared with other treatment techniques, AOPs could effectively eliminate organic compounds in the aqueous phase, rather than accumulating or transferring pollutants into another phase. Due to the remarkable reactivity of the •OH radical, AOPs could therefore be applicable in many, if not all, scenarios where many organic contaminants are expected to be removed at the same time.

Sorption is another process used to mitigate organic pollutants, and many materials have been applied towards designing permeable reactive barriers (PRBs), a cost-effective technology for in situ groundwater remediation [7]. In these permeable reactive barriers, the sorption [8] and chemical oxidation reactions [9] are the main processes, accompanied to a less extent by precipitation and biochemical reactions. Therefore, there is a need for new materials able to perform both adsorption and AOPs of refractory toxic chemicals.

MOFs comprise inorganic nodes (e.g., chains, clusters, atoms) and various organic linkers (e.g., phosphonates, azolates, carboxylates, etc.) that can form multidimensional periodic lattices. They represent a novel class of porous and permeable coordination polymers [10]. One of the most important characteristics is their large and accessible surface area up to 7000 m^2^/g, as well their low pore volumes. For the formation of MOF structures, organic molecules called bridging ligands attach to the secondary building units (SBU) [11]. For the synthesis of MOFs, the typically used bridging ligands are di- and tricarboxylic acids, such as 1,4-benzene dicarboxylic acid (also called terephthalic acid), biphenyl-4,4′-dicarboxylic acid (BPDC), and benzene-1,3,5-tricarboxylic acid (trimesic acid) [10]. In recent years, a wide range of MOFs [12] has been used in environmental applications including processes such as adsorption, photocatalysis, and microwave catalysis [13]. These different MOFs show a synergistic mixture of features, since they have the shape of building units and their chemical compositions multiply based on targeted structures [14].

MOFs are promising materials for the removal of contaminants from water [15,16], since they exhibit numerous properties that enable their use in water treatment [17]: (i) they have a certain stability in water; (ii) they have high sorption capacities, attributable to large specific surface area and pore volume; (iii) they have openings for adjusting their structure to allow for shape-selective adsorption and/or catalysis; (iv) they have active sites, where contaminants can be adsorbed or transformed; (v) they have functionalizable cavities, where host−guest interactions can occur; (vi) the synthesis of some MOFs can be scaled-up; and (vii) they can be molded as monoliths, pellets, membranes, or columns, that can be used in degradation reactors.

The objective of this study is to provide a comprehensive and novel overview of the MOFs used in adsorption and catalysis processes for the removal of priority and emerging organic contaminants from wastewater.

## 2. Types of Environmentally Relevant MOFs

### 2.1. Nomenclature of MOFs

In order to describe and organize the structures of MOF-type compounds, a nomenclature system has been developed. The subunit of a MOF, called the secondary construction unit (SBU), can be described by topologies similar to those of other structures. Each topology, also called network/net, is assigned to a symbol consisting of 3–5 letters followed by a number. IRMOF-1 (more commonly called MOF-5) and IRMOF-16 belong to the same MOF families, having the same type of molecular symmetry [18]. Many MOFs were named after the site of discovery and other MOFs have similar names to zeolites (Table 1).

### 2.2. Stability of MOFs in Aquatic Environment

**Figure 1:**
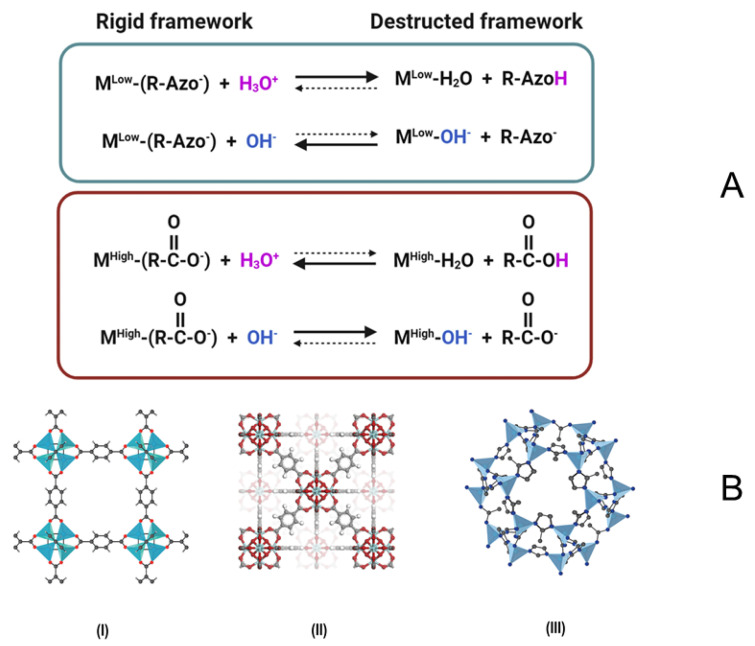
Metal–ligand substitution reactions between MOFs and protons/hydroxides M^High^/M^Low^, high-/low-valence metal nodes. Solid arrows represent a more favored process, and dashed arrows represent a less favored process. Adapted with permission from Ref. [13] 2021, Elsevier (**A**). The crystal structure diagrams for MOF-5 (I), UiO-66 (II), and ZIF-8 (III) [44] (**B**).

The feasibility of MOFs as adsorption materials and catalysts in the removal of organic pollutants depends on their chemical stability under various environmental conditions, which is determined by the ability of MOFs to maintain their long-range ordered structures in water, especially in acidic and alkaline conditions [40]. In certain conditions, water and other polar solvents can damage the MOF structures either by affecting the metal–ligand bonds or by metal ion solvation. Due to their strong coordination tendency, some strong nucleophilic reagents that might be present in water, such as amines, hydroxides, and alkoxides, also tend to replace the ligand and disintegrate MOF structures [41]. MOFs containing weak metal–ligand bonds tend to undergo hydrolysis or ligand displacement or in water [13]. MOFs with soft acid metals and soft base ligands generally exhibit high stability under alkaline conditions and low stability under acidic conditions, while those with hard acid metals and hard base ligands experience the opposite trend [42] (Figure 1A). At low pH values, protons compete with metal ions for coordination with organic ligands. Since soft base ligands have relatively high pK_a_ values, they tend to remain protonated at a low pH and have a high affinity for protons. Therefore, protons are more likely to compete with and substitute metal ions, causing the MOF structure to decompose. On the contrary, the pK_a_ of hard base ligands is relatively low, as these ligands deprotonate even at a low pH and have a low affinity for protons [13]. It was found that the UiO-66 maintained its stability in water for about 12 months during the adsorption of methyl orange [43].

In general, crystal shape of a MOF is related to its internal structure (see Figure 1B for MOF-5 [45], UiO-66, and ZIF-8) [44].

Nevertheless, a smaller number of MOFs have been applied in the removal of organic contaminants from wastewater. An overview summarizing the types of MOFs, the targeted contaminants, and the performance of the relevant adsorption and catalysis processes can be found in Table 2.

In this respect, there is great attention paid to designing MOFs for the adsorption process in order to remove pollutants, because it is straightforward, simplistic, practical, and inexpensive, and it can be used for an extensive range of organic pollutants.

Another direction of research is the use of MOFs in catalysis processes for the degradation of toxic organic pollutants. In this respect, in order to advance the guidelines for optimum synthesis of MOF catalysts, determining the dynamics of MOFs and their catalytic sites, as well as the intrinsic kinetics of the catalytic reaction, is needed.

## 3. Removal of Priority and Emerging Organic Contaminants from Wastewater by Adsorption Processes Using MOFs

**Figure 2:**
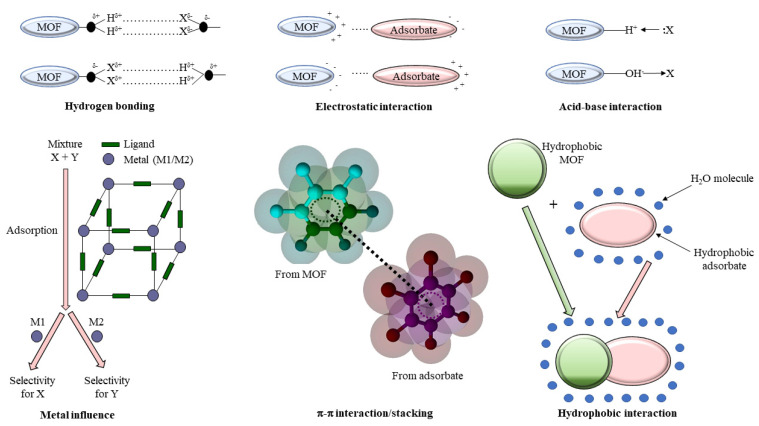
Adsorptive removal of CECs by MOFs based on different interactions [1].

In order to design new MOFs to study the adsorption of priority and emerging organic contaminants, a thorough understanding of the adsorption mechanism is needed. Usually, the adsorption mechanism of organic contaminants by MOFs is determined by hydrophobic interactions, π−π interactions, acid−base interactions, electrostatic interactions, and hydrogen bonding or a combination of these factors (Figure 2). Aside from the nature of adsorbed contaminants, the surface area and porosity of the MOF also play a crucial role in the adsorption process [57].

**Figure 3:**
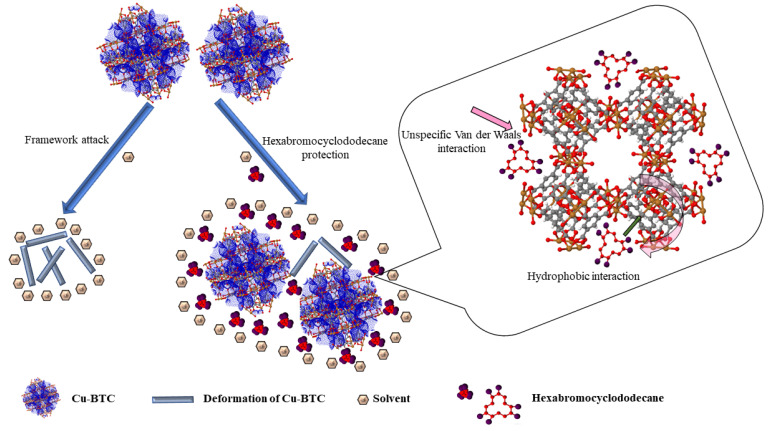
Adsorption mechanism of HBCD onto Cu-BTC [46].

The hydrophobic interactions are dominant in the case of hydrophobic organic contaminants with values of octanol participation coefficients higher than six (log *K*_ow_ > 6). The π−π interactions/stackings are relevant in the case of organic contaminants with planar structures (polycyclic aromatic hydrocarbons (PAHs) [58], co-planar polychlorinated biphenyls (PCBs) [59], polychlorinated dibenzo-p-dioxins and polychlorinated dibenzo-p-furans (PCDDs/Fs) [60], and polybrominated dibenzo-p-dioxins and dibenzofurans (PBDDs/Fs) [61], etc.), while acid−base interactions with MOF structures are relevant in the case of organic contaminants with acid−base properties (possibly for chlorophenols and bromophenols). The removal of hexabromocyclododecane (HBCD) from an aquatic environment was studied using two Cu- and Fe-based metal–organic frameworks (Cu-BTC and Fe-BTC) [46]. It was observed that over 80% of HBCD was removed by Cu-BTC within 5 h [47]. The adsorption mechanism involved van der Waals and hydrophobic interactions (Figure 3).

The reported maximum adsorption capacities of Cu-BTC and Fe-BTC were 39 and 21.5 mg/g pH = 7 [47], respectively. Two MIL-101-based metal–organic frameworks (Cr-MIL-101 and Fe-MIL-101-NH_2_) were used in the adsorption of the emerging contaminant triphenyl phosphate (TPhP), suggesting that hydrophobic interactions may play a dominant role in the selective adsorption process of TPhP, while π–π interactions may also be significantly weak [48]. The maximum adsorption capacities of Cr-MIL-101 and Fe-MIL-101-NH_2_ were also compared with those of activated carbon and they were found to decrease in the order of Cr-MIL-101 > Fe-MIL-101-NH_2_ > activated carbon, while the initial sorption velocities(v_0_) increased in the order of 105.04 μmol/g·h for activated carbon < 170.36 μmol/g·h for Fe-MIL-101-NH2 < 568.18 μmol/g·h for Cr-MIL-101. It was found that the TPhP adsorption equilibrium on Cr-MIL-101 was reached within 12 h, while the equilibrium time on Fe-MIL-101-NH_2_ was reached in about 48 h. The initial adsorption velocity of TPhP on Cr-MIL-101 was 568.18 μmol/gL, significantly faster than those of Fe-MIL-101-NH_2_ and activated carbon [47]. Remarkably, investigations into the effect of ion strength found that when the Ca^2+^ concentration was raised from 0 to 5 mmol/L, the TPhP removal efficiency on Cr-MIL-101 increased from 59.1% to 90.2% [48].

Two iron-based MOFs [MIL-88(Fe) and NH_2_-MIL-88(Fe)] were used to remove pyrene from water [48]. It was found that the pyrine adsorption isotherm was best explained by the Langmuir model, while the kinetics of the adsorption were found to follow the pseudo-second-order model [48]. The removal efficiency of pyrine after 40 min was calculated, achieving 96.0% in NH_2_-MIL-88(Fe) and 99.7% in MIL-88(Fe), respectively, suggesting that these two MOFs can serve as possible adsorbents for the removal of PAHs from wastewater [48]. The adsorption of 2-chlorophenol (2-CP) by MIL-101 and its amino-derivative (MIL-101-NH_2_) was investigated in a batch adsorption study [49]_._ The equilibrium uptake of 2-CP after 24 h was 121 mg/g for MIL-101 and 84 mg/g for MIL-101-NH_2_, respectively, lower than the value of 345 mg/g recorded in the same study for activated carbon [49], while the removal efficiency of 2-CP by MIL-101 was about 60%.

## 4. Removal of Priority and Emerging Organic Contaminants from Wastewater by Catalysis Processes Using MOFs

Due to their simplicity and reproducibility, advanced catalysis processes involving MOFs (photocatalysis, AOPs, etc.) have emerged as new methods to remove organic pollutants from wastewater [62]. In order to enhance the oxidation of organic pollutants into less toxic products or to their complete mineralization, the radicals (OH• or SO_4_•^−^) are produced using highly reactive compounds (such as hydrogen peroxide (H_2_O_2_) and persulfate salts (i.e., K_2_S_2_O_8_), in combination with MOFs [63]. The role of MOFs in degradation of CECs is based on two main properties: (i) MOFs can become catalytically active upon modification; and (ii) MOFs can exhibit intrinsic catalytic activity.

As an environmentally friendly oxidant, H_2_O_2_ has long been widely used in various water purification processes, and various AOPs have been developed. Because of the unique structure and excellent performance of MOFs, it has been confirmed through many experiments that the introduction of MOFs into the Fenton system can overcome the drawbacks of traditional Fenton-like methods. Many typical organic contaminants such as organic dyes [64], phenols [65], and acid orange 7 (AO7) [66] are known to be degraded in a Fenton-like system catalysed by MOFs. 

### 4.1. Fenton Processes Involving MOFs

The H_2_O_2_-based Fenton removal of high concentrations of methylene blue in wastewater (500 pm) was studied using MIL-100(Fe) and FeII@MIL-100(Fe) with high surface areas (1646 m^2^/g and 1228 m^2^/g, respectively) [50]. An increased Fenton catalytic ability was found in Fe^II^@MIL-100(Fe), compared with MIL-100(Fe) and Fe_2_O_3_ catalyst activities. Nevertheless, the total removal efficiency was found to be around 97% in MIL-100(Fe), 90% in Fe^II^@MIL-100(Fe), and around 60% in Fe_2_O_3_ after a total time of 285 min [50]. This was attributed both to Fenton oxidation as well as to adsorption via electrostatic interactions between negative (or positive) adsorbent and positively charged methylene blue [50].

**Figure 4:**
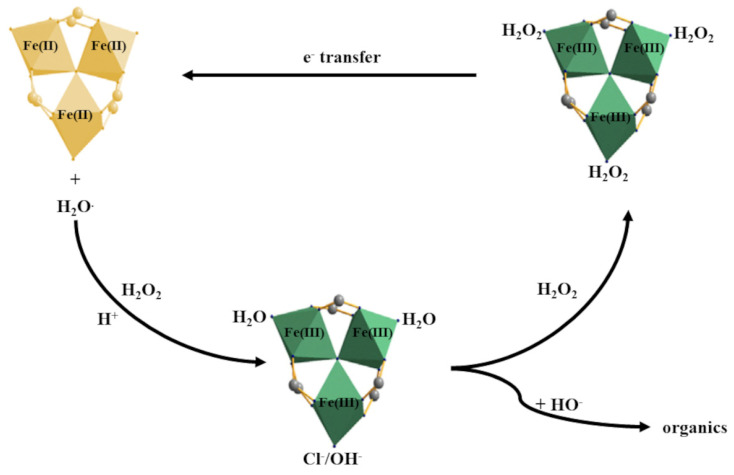
The pathway of H_2_O_2_ activation by MIL-88B-Fe for oxidation of organic pollutants [52].

With respect to the aromatic compounds, the H_2_O_2_-based Fenton oxidation of phenol was investigated in a batch glass reactor using MIL-53(Fe) as the catalyst in two successive studies [51]. Both studies found that MIL-53(Fe) leads to an over 90% degradation of phenol at neutral pH values after 3 h (Table 2). Nevertheless, a better degradation rate (99%) after 30 min of reaction was obtained using the same H_2_O_2_-based Fenton system but with MIL-88B-Fe as a catalyst [52]. Based on this reaction, a new degradation pathway of organic pollutants in the H_2_O_2_-based Fenton system was proposed, using activation with MIL-88B-Fe. This mechanism suggests that the Fe(III) was converted to Fe(II) due to charge transfer after H_2_O_2_ reached the active sites, while the Fe(II) reacted with H_2_O_2_ to generate •OH and degraded organic pollutants, like in the classical Fenton systems (Figure 4) [52].

### 4.2. Electrocatalysis Processes Involving MOFs

**Figure 5:**
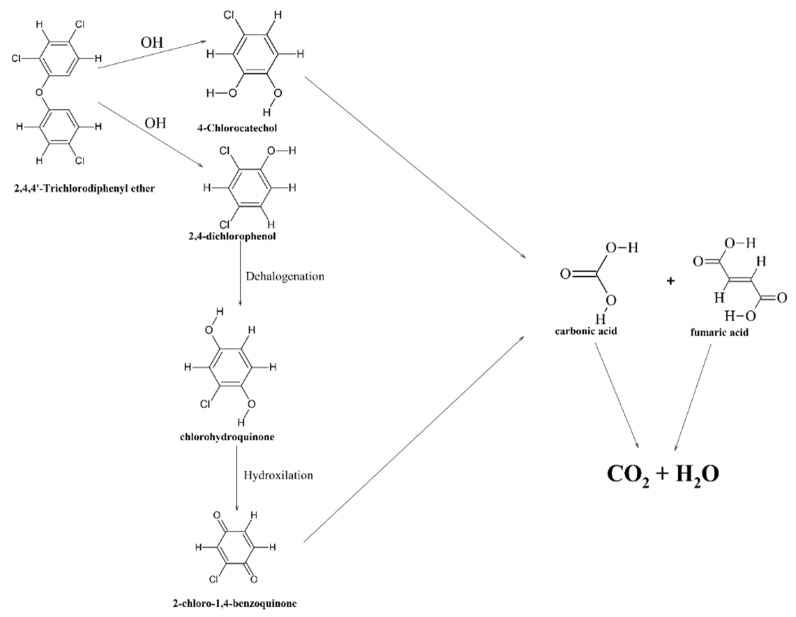
The proposed pathways of the degradation of TCS in a hetero-electro-Fenton process with the Mn/Fe@PC-CP cathode according to [53].

Mn-doped MIL-53(Fe) was used as the cathode material for the electro-Fenton catalysis of the emerging contaminant triclosan (TCS), and the highest TCS removal efficiency (about 99.9 ± 0.1%) was obtained with the Mn/Fe@PC-CP cathode at a rate constant (*k*) of 0.06 min^−1^ [53]. A degradation pathway was proposed: TCS was adsorbed and enriched on the surface of the hydrophobic cathode, after which the phenolic ring of TCS was attacked by reactive oxygen species, such as •OH on the C(1)- or C(2)- position of TCS, and converted to 2,4-dichlorophenol (2,4-DCP) and 4-chlorocatechol (4-CC) (Figure 5) [53].

Afterwards, the chloro-*p*-benzoquinone was produced by hydroxylation and dehalogenation of 2,4-dichlorophenol by •OH on the C(4)-position. Finally, the intermediates were oxidized into small molecular substances such as carboxylic acids, which mineralized into CO_2_ and H_2_O.

ZIF-67 was involved in the synthesis of several iron and cobalt dual metal- and nitrogen-doped carbons (FeCoNCs) as the electrocatalyst for cathode materials in the oxygen reduction reaction from microbial fuel cells (MFCs), devices that can be used in the biochemical degradation of organic pollutants [67]. FeCoNC materials showed excellent durability and stability as the cathode oxygen reduction catalyst; the maximum power density of the FeCoNC-modified air–cathode microbial fuel cell (MFC) was recorded as 1769.95 mW/m^2^, higher than that of Pt/C-modified MFC (1410.31 mW/m^2^) [67].

Recently, metal–organic framework-derived iron-oxide-modified carbon cloth (MIL-88(Fe)) was applied as a high-power-density microbial fuel cell anode [68]. The MFC containing the MIL-Fe_3_O_4_/CC anode recorded a power density of 4305 mW/m^2^ [69]. 

### 4.3. Photocatalysis Processes Involving MOFs

**Figure 6:**
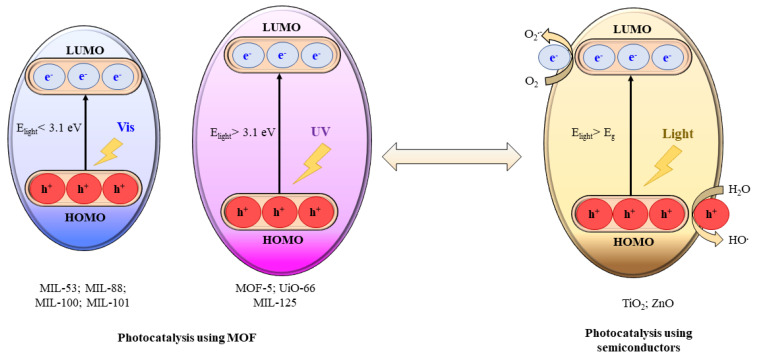
Mechanism of MOF photocatalysis vs. semiconductor photocatalysis. Comparison of band gaps and light source (UV or visible light) between representative MOFs [69].

Based on the principle of traditional semiconductor photocatalysis, a MOF photocatalyst can be directly excited by incident light with energy (E_light_) larger than the band gap (E_g_). In this way, electron–hole (e^−^–h^+^) pairs can be generated (Figure 6), creating hydroxyl radicals (HO^•^) [57]. Due to the occurrence of reactive species (O^2•−^, HO^•^ and h^+^), various legacy and emerging organic contaminants can be oxidized [69].

As mentioned earlier, the photocatalysis processes induced by MOFs were efficient in the removal of organic dyes and phenols [69,70]. For example, Cu(4,4′-bipy)Cl]*_n_* (1) and [Co(4,4′-bipy)·(HCOO)_2_]*_n_* (2) were applied for the photocatalytic degradation of methylene blue in the H_2_O_2_/UV system [54]. After four cycles, the complex (1) retained 83.18% of methylene blue, while the other MOF complex (2) retained 67.83% of methylene blue [54] (Table 2). Fe-BTC was used in a batch reactor for the photocatalytic oxidation of phenol to dihydroxybenzenes (DHBZ) in the H_2_O_2_/UV system [70]. The photocatalytic oxidation of phenol in the presence of Fe-BTC resulted in a high DHBZ selectivity (65%) and yield (35%), higher than those obtained for other Fe-based MOFs. A novel Mn-doped Fe-based metal–organic framework (MOF) was synthesized recently [55] based on MIL-88B-Fe for the removal of phenol using Fenton-like and photo-Fenton reactions. A maximum degradation efficiency of 96% was obtained during photocatalysis at 8 wt.% in Mn-doped MIL-88-Fe [55].

Nevertheless, regarding the photocatalysis of the compounds included in the Stockholm Convention of Persistent Organic Pollutants (POPs), a small number of MOFs were recorded as efficient photocatalysts [57]. For example, the dechlorination of 1,1-bis(4-chlorophenyl)-2,2,2-trichloroethane (DDT) under visible light irradiation was catalyzed by B12–Ru@MOF (where the basic MOF is [Zn_4_Ru_2_(bpdc)_4_·4C_2_NH_8_·9DMF]_n_) and under reductive condition (without H_2_O_2_), resulting in yields of 99% (see Table 2) and 63% for the transformation of DDT to 1,1-bis(4-chlorophenyl)-2,2-dichloroethane after 4 h in the first and third cycles, respectively [56].

## 5. Challenges in Using MOFs for the Removal of Priority and Emerging Organic Contaminants from Wastewater

By far, the main challenge in the removal of priority and emerging organic contaminants from wastewater by adsorption is transferring the pollutant in another phase. Therefore, the treatment of large amounts of wastewater by adsorption on MOFs seems to be problematic. Nevertheless, in the case of permeable reactive barriers (PRBs), MOFs appear to be a good solution to immobilize organic pollutants, while adsorption can also be combined with other processes such as chemical oxidation and biological degradation, leading to the degradation of organic contaminants.

Various studies have shown that it is relatively complicated to establish general trends on how the properties of CECs influence their removal when MOFs are used, due to the variation of physicochemical properties amongst CECs. Nevertheless, among the potential mechanisms for CEC removal, these main mechanisms are influential in the following sequence: electrostatic interactions > binding interactions > stacking interactions > hydrophobic interactions ≈ acid–base interactions ≈ metallic effect [1]. Moreover, these interactions can change depending on the water chemistry at that moment. One of the main concerns regarding adsorption, in terms of environmental sustainability, consists in water reproducibility, which can be ameliorated by mixing in various cleaning agents.

Further development of MOF synthesis methods is required, because their physicochemical characteristics are crucially influenced by reaction time, particle size, and morphology. As a consequence, quantitative structure–activity relationship investigation may be needed to explain the adsorption mechanisms for CECs on various MOFs, in order to understand the interactions between the functional groups of the CECs whose molecules having the highest activity. Most MOFs are produced as powder, so an ultrafiltration process must be used to recover them from water after adsorption and catalysis treatments. Moreover, to become competitive with commercial adsorbents available for water and wastewater treatment, MOFs must be cost-effective and cheap to produce. In addition, more studies are necessary to assess the ecotoxicological impacts of MOFs, especially when they are to be disposed.

The use of MOFs in Fenton processes is considered a challenge when applied to highly halogenated organic compounds (HCHs, HBCDs, etc.), and as a result those technologies might not be useful in waters with high bicarbonate content [71,72,73], which is the same problem as the case of classical Fenton processes. The photocatalytic processes involving MOFs are the most promising for the removal of priority and emerging organic contaminants from wastewater, but they should be further tested on a larger number of compounds and on higher concentrations of pollutants.

## 6. Conclusions

MOFs can be considered as promising materials both in adsorption processes and catalysis, mainly in AOPs. Nevertheless, their costs are still high compared to other adsorbents (i.e., activated carbon) or catalysts (Fe_2_O_3_, TiO_2_, etc.). In this respect, in order to decrease production costs, further studies to develop more efficient synthesis methods must be completed.

However, MOFs exhibit promising incorporation in wastewater treatment at the industrial scale. The tunability of their structural and electronic characteristics results in the efficient preparation of materials that are good adsorbents as well as efficient Fenton and photo-Fenton catalysts. In addition, the use of eco-friendly ligands (like fumaric, maleic and succinic acids) opens up the possibility of using MOF materials to design permeable reactive barriers (PRBs) as a cost-effective technology for in situ groundwater remediation. Furthermore, the prospect of extensive use of MOFs in wastewater treatment is encouraging but still needs further exploration, in terms of scaling up their application in real conditions. Further challenges and perspectives include the occurrence of new emerging contaminants in the next years, as well as their possible incomplete degradation in MOF-catalyzed processes leading to the formation of toxic degradation products. This review summarizes the fundamentals of adsorption and photodegradation as common applications in MOFs for the removal of legacy and emerging organic contaminants from wastewater. In short, MOFs can be regarded as one of the hottest topics of research today in material and environmental science. Due to their promising properties, MOFs are very tempting materials for researchers to further explore and investigate the removal of legacy and emerging contaminants from wastewater.

## Figures and Tables

**Table 1:** Example of typical names for metal–organic structures (MOF) with the description of their molecular formulas.

No.	Name	Molecular Formula	Reference	Abbreviation Legend
1	IRMOF-1 or MOF-5	Zn_4_O(BDC)_3_. 7DEF.3H_2_O	[18]	IsoReticular metal–organic frameworks
2	IRMOF-16	Zn_4_O(TPDC)_3_. 17DEF.2H_2_O	[19]	
3	CPL-2	Cu_2_(PZDC)_2_(4,4′-BPY)	[20]	Coordination polymers with a pillared layer structure
4	F-MOF-1	[Cu(HFBBA)(phen)_2_](H_2_HFBBA)_2_(H_2_O)(HCO_2_)	[21]	Fluorinated metal–organic framework
5	MOP-1	Cu_24_(m-BDC)_24_(DMF)_14_(H_2_O)_10_	[22]	Metal–organic polyhedra
6	HKUST-1 (MOF-199)	Cu_3_(BTC)_2_	[23]	Hong Kong University of Science and Technology
7	LIC-1	Gd_2_(BDC-NH_2_)_3_(DMF)_4_	[24]	Leiden Institute of Chemistry
8	ZIF-8	Zn(MIM)_2_	[25]	Zeolite imidazolate framework
9	ZIF-90	Zn(FIM)_2_	[26]	
10	MOF-74	Zn_2_DOT	[23]	Metal–organic frameworks
11	MOF-101	Cu_2_(BDC-Br)_2_(H_2_O)_2_	[27]	
12	MOF-177	Zn_4_O(BTB)_2_	[23]	
13	MOF-235	[Fe_3_O(BDC)_3_(DMF)_3_][FeCl_4_].(DMF)_3_	[28]	
14	MOF-253	Al(OH)(BPYDC)	[29]	
15	UiO-66	Zr_6_O_6_(BDC)_6_	[30]	Universitetet i Oslo
16	UiO-67	Zr_6_O_6_(BPDC)_6_	[31]	
17	UiO-68	Zr_6_O_6_(TPDC)_6_	[32]	
18	MIL-53	Al(OH)(BDC)	[33]	Materials of Institut Lavoisier
19	MIL-53(Al)-NH_2_	Al(OH)(BDC-NH_2_)	[34]	
20	MIL-88A	Fe_3_O(MeOH)_3_(O_2_CCH=CHCO_2_)_3_.MeCO_2_.*n*H_2_O	[35]	
21	MIL-88-Fe	Fe_3_O(MeOH)_3_(O_2_C(CH_2_)_2_CO_2_)_3_. AcO.(MeOH)_4.5_	[36]	
22	MIL-88B-4CH3	2Fe_3_O(OH)(H_2_O)_2_(BDC-Me_2_)_3_	[37]	
23	MIL-100-Fe	Fe^III^_3_ O(H_2_O)_2_F.(BTC)_2_. *n*H_2_O	[38]	
24	MIL-101	Cr_3_O(H_2_O)_2_F.(BDC)_3_. *n*H_2_O	[39]	

Ligand abbreviations: m-BDC = m-benzenedicarboxylate, TPDC = p-terphenyl-4,4′-dicarboxylate, PZDC = pyrazine-2,3-dicarboxylate, HFBBA = 4,4- hexafluoroisopropylidene)dibenzoate, MIM = 2-methylimidazolate, DOT 2,5-dihydroxyterephthalate, BPYDC = 2,2′-bipyridine-5,5′-dicarboxylate, BPDC = biphenyl-4,4′-dicarboxylate, DEF = N,N-diethylformamide, FIM = 2-formylimidazolate, 4,4′-BPY = 4,4′-bipyridine, phen = 1,10-phenanthroline.

**Table 2:** Summary of adsorption and catalysis studies for relevant priority and emerging organic contaminants.

No.	Name of MOF	Target Contaminant	Reference	Type of Process	Performance of Adsorption/Catalysis Processes
1	Cu-BTC and Fe-BTC	HBCD	[46]	Adsorption	Over 80% of HBCD removed by Cu-BTC
2	Cr-MIL-101 and Fe-MIL-101-NH_2_	TPhP	[47]	Adsorption	Removal efficency of 90.2% by Cr-MIL-101
3	MIL-88(Fe) and NH_2_-MIL-88(Fe)	Pyrine	[48]	Adsorption	Removal efficency of 96.0% for NH_2_-MIL-88(Fe) and 99.7% for MIL-88(Fe))
4	MIL-101 and MIL-101-NH_2,_	2-chlorophenol (2-CP)	[49]	Adsorption	Removal efficiency of 60% on MIL-101
5	MIL-100(Fe) and FeII@MIL-100(Fe)	Methylene blue	[50]	Fenton	Removal efficiency of around 96% -for MIL-100(Fe) and and 90% Fe^II^@MIL-100(Fe)
6	MIL-53(Fe)	Phenol	[51]	Fenton	90% degradation
7	MIL-88B-Fe	Phenol	[52]	Fenton	99% degradation
8	Mn-doped MIL-53(Fe)	TCS	[53]	Electrocatalysis	Removal efficency of 99.9 ± 0.1%
9	Cu(4,4′-bipy)Cl]*_n_* (**1**) and [Co(4,4′-bipy)·(HCOO)_2_]*_n_ (2)*	Methylene blue	[54]	Photocatalysis	Removal efficiency of 83.18% for Cu(4,4′-bipy)Cl]*_n_* (**1**) and of 67.83% [Co(4,4′-bipy)·(HCOO)_2_]*_n_ (2)*
10	Mn-doped MIL-88-Fe	Phenol	[55]	Photocatalysis	Removal efficency of 96%
11	B12–Ru@[Zn_4_Ru_2_(bpdc)_4_·4C_2_NH_8_·9DMF]_n_	DDT	[56]	Photocatalysis	Transformation yield of 99%

## Data Availability

Not applicable.

## References

[1] Joseph L., Jun B.-M., Jang M., Park C.M., Muñoz-Senmache J.C., Hernández-Maldonado A.J., Heyden A., Yu M., Yoon Y. (2019). Removal of Contaminants of Emerging Concern by Metal-Organic Framework Nanoadsorbents: A Review. Chem. Eng. J..

[2] Sengupta A., Jebur M., Kamaz M., Wickramasinghe S.R. (2022). Removal of Emerging Contaminants from Wastewater Streams Using Membrane Bioreactors: A Review. Membranes.

[3] Barbosa M.O., Moreira N.F.F., Ribeiro A.R., Pereira M.F.R., Silva A.M.T. (2016). Occurrence and Removal of Organic Micropollutants: An Overview of the Watch List of EU Decision 2015/495. Water Res..

[4] Wilhelm S., Henneberg A., Köhler H.-R., Rault M., Richter D., Scheurer M., Suchail S., Triebskorn R. (2017). Does Wastewater Treatment Plant Upgrading with Activated Carbon Result in an Improvement of Fish Health?. Aquat. Toxicol..

[5] Bokare A.D., Choi W. (2014). Review of Iron-Free Fenton-like Systems for Activating H_2_O_2_ in Advanced Oxidation Processes. J. Hazard. Mater..

[6] Lee B.C., Lim F.Y., Loh W.H., Ong S.L., Hu J. (2021). Emerging Contaminants: An Overview of Recent Trends for Their Treatment and Management Using Light-Driven Processes. Water.

[7] Abbas T., Wadhawan T., Khan A., McEvoy J., Khan E. (2021). Iron Turning Waste: Low Cost and Sustainable Permeable Reactive Barrier Media for Remediating Dieldrin, Endrin, DDT and Lindane in Groundwater. Environ. Pollut..

[8] Ma J., Wang Y., Stevens G.W., Mumford K.A. (2020). Hydrocarbon Adsorption Performance and Regeneration Stability of Diphenyldichlorosilane Coated Zeolite and Its Application in Permeable Reactive Barriers: Column Studies. Microporous Mesoporous Mater..

[9] Gholami F., Mosmeri H., Shavandi M., Dastgheib S.M.M., Amoozegar M.A. (2019). Application of Encapsulated Magnesium Peroxide (MgO2) Nanoparticles in Permeable Reactive Barrier (PRB) for Naphthalene and Toluene Bioremediation from Groundwater. Sci. Total Environ..

[10] Raptopoulou C.P. (2021). Metal-Organic Frameworks: Synthetic Methods and Potential Applications. Materials.

[11] Park S.S., Hendon C.H., Fielding A.J., Walsh A., O’Keeffe M., Dincă M. (2017). The Organic Secondary Building Unit: Strong Intermolecular π Interactions Define Topology in MIT-25, a Mesoporous MOF with Proton-Replete Channels. J. Am. Chem. Soc..

[12] Yu S., Pang H., Huang S., Tang H., Wang S., Qiu M., Chen Z., Yang H., Song G., Fu D. (2021). Recent Advances in Metal-Organic Framework Membranes for Water Treatment: A Review. Sci. Total Environ..

[13] Wen Y., Zhang P., Sharma V.K., Ma X., Zhou H.-C. (2021). Metal-Organic Frameworks for Environmental Applications. Cell Rep. Phys. Sci..

[14] Safaei M., Foroughi M.M., Ebrahimpoor N., Jahani S., Omidi A., Khatami M. (2019). A Review on Metal-Organic Frameworks: Synthesis and Applications. TrAC Trends Anal. Chem..

[15] Ahmadijokani F., Molavi H., Rezakazemi M., Tajahmadi S., Bahi A., Ko F., Aminabhavi T.M., Li J.-R., Arjmand M. (2022). UiO-66 Metal–Organic Frameworks in Water Treatment: A Critical Review. Prog. Mater. Sci..

[16] Oladoye P.O., Adegboyega S.A., Giwa A.-R.A. (2021). Remediation Potentials of Composite Metal-Organic Frameworks (MOFs) for Dyes as Water Contaminants: A Comprehensive Review of Recent Literatures. Environ. Nanotechnol. Monit. Manag..

[17] Rojas S., Horcajada P. (2020). Metal–Organic Frameworks for the Removal of Emerging Organic Contaminants in Water. Chem. Rev..

[18] Yaghi O.M., O’Keeffe M., Ockwig N.W., Chae H.K., Eddaoudi M., Kim J. (2003). Reticular Synthesis and the Design of New Materials. Nature.

[19] Yuksel N., Kose A., Fellah M.F. (2022). A DFT Investigation of Hydrogen Adsorption and Storage Properties of Mg Decorated IRMOF-16 Structure. Colloids Surf. A Physicochem. Eng. Asp..

[20] Xiang H., Ameen A., Shang J., Jiao Y., Gorgojo P., Siperstein F.R., Fan X. (2020). Synthesis and Modification of Moisture-Stable Coordination Pillared-Layer Metal-Organic Framework (CPL-MOF) CPL-2 for Ethylene/Ethane Separation. Microporous Mesoporous Mater..

[21] Yang C., Wang X., Omary M.A. (2007). Fluorous Metal−Organic Frameworks for High-Density Gas Adsorption. J. Am. Chem. Soc..

[22] Guillerm V., Kim D., Eubank J.F., Luebke R., Liu X., Adil K., Lah M.S., Eddaoudi M. (2014). A Supermolecular Building Approach for the Design and Construction of Metal–Organic Frameworks. Chem. Soc. Rev..

[23] Tranchemontagne D.J., Hunt J.R., Yaghi O.M. (2008). Room Temperature Synthesis of Metal-Organic Frameworks: MOF-5, MOF-74, MOF-177, MOF-199, and IRMOF-0. Tetrahedron.

[24] Venkata Sravani V., Sengupta S., Sreenivasulu B., Gopakumar G., Tripathi S., Chandra M., Brahmmananda Rao C.V.S., Suresh A., Nagarajan S. (2022). Highly Efficient Functionalized MOF-LIC-1 for Extraction of U(vi) and Th(Iv) from Aqueous Solution: Experimental and Theoretical Studies. Dalton Trans..

[25] Bergaoui M., Khalfaoui M., Awadallah-F A., Al-Muhtaseb S. (2021). A Review of the Features and Applications of ZIF-8 and Its Derivatives for Separating CO2 and Isomers of C3- and C4- Hydrocarbons. J. Nat. Gas Sci. Eng..

[26] Liu C., Liu Q., Huang A. (2016). A Superhydrophobic Zeolitic Imidazolate Framework (ZIF-90) with High Steam Stability for Efficient Recovery of Bioalcohols. Chem. Commun..

[27] Eddaoudi M., Kim J., O’Keeffe M., Yaghi O.M. (2002). Cu_2_[o-Br-C_6_H_3_(CO_2_)_2_]_2_(H_2_O)_2_·(DMF)_8_(H_2_O)_2_: A Framework Deliberately Designed to Have the NbO Structure Type. J. Am. Chem. Soc..

[28] Li Y., Hou G., Yang J., Xie J., Yuan X., Yang H., Wang M. (2016). Facile Synthesis of MOF 235 and Its Superior Photocatalytic Capability under Visible Light Irradiation. RSC Adv..

[29] Valvekens P., Bloch E.D., Long J.R., Ameloot R., De Vos D.E. (2015). Counteranion Effects on the Catalytic Activity of Copper Salts Immobilized on the 2,2′-Bipyridine-Functionalized Metal–Organic Framework MOF-253. Catal. Today.

[30] Winarta J., Shan B., Mcintyre S.M., Ye L., Wang C., Liu J., Mu B. (2020). A Decade of UiO-66 Research: A Historic Review of Dynamic Structure, Synthesis Mechanisms, and Characterization Techniques of an Archetypal Metal–Organic Framework. Cryst. Growth Des..

[31] Kaur G., Øien-Ødegaard S., Lazzarini A., Chavan S.M., Bordiga S., Lillerud K.P., Olsbye U. (2019). Controlling the Synthesis of Metal–Organic Framework UiO-67 by Tuning Its Kinetic Driving Force. Cryst. Growth Des..

[32] Ye X., Liu D. (2021). Metal–Organic Framework UiO-68 and Its Derivatives with Sufficiently Good Properties and Performance Show Promising Prospects in Potential Industrial Applications. Cryst. Growth Des..

[33] Nguyen D.T.C., Le H.T.N., Do T.S., Pham V.T., Lam Tran D., Ho V.T.T., Tran T.V., Nguyen D.C., Nguyen T.D., Bach L.G. (2019). Metal-Organic Framework MIL-53(Fe) as an Adsorbent for Ibuprofen Drug Removal from Aqueous Solutions: Response Surface Modeling and Optimization. J. Chem..

[34] Huang L., Yang Z., Li X., Hou L., Alhassan S.I., Wang H. (2021). Synthesis of Hierarchical Hollow MIL-53(Al)-NH2 as an Adsorbent for Removing Fluoride: Experimental and Theoretical Perspective. Environ. Sci. Pollut. Res..

[35] Andrew Lin K.-Y., Chang H.-A., Hsu C.-J. (2015). Iron-Based Metal Organic Framework, MIL-88A, as a Heterogeneous Persulfate Catalyst for Decolorization of Rhodamine B in Water. RSC Adv..

[36] Zango Z.U., Jumbri K., Sambudi N.S., Hanif Abu Bakar N.H., Fathihah Abdullah N.A., Basheer C., Saad B. (2019). Removal of Anthracene in Water by MIL-88(Fe), NH2-MIL-88(Fe), and Mixed-MIL-88(Fe) Metal–Organic Frameworks. RSC Adv..

[37] Horcajada P., Salles F., Wuttke S., Devic T., Heurtaux D., Maurin G., Vimont A., Daturi M., David O., Magnier E. (2011). How Linker’s Modification Controls Swelling Properties of Highly Flexible Iron(III) Dicarboxylates MIL-88. J. Am. Chem. Soc..

[38] Mahmoodi N.M., Abdi J., Oveisi M., Alinia Asli M., Vossoughi M. (2018). Metal-Organic Framework (MIL-100 (Fe)): Synthesis, Detailed Photocatalytic Dye Degradation Ability in Colored Textile Wastewater and Recycling. Mater. Res. Bull..

[39] Du P.D., Thanh H.T.M., To T.C., Thang H.S., Tinh M.X., Tuyen T.N., Hoa T.T., Khieu D.Q. (2019). Metal-Organic Framework MIL-101: Synthesis and Photocatalytic Degradation of Remazol Black B Dye. J. Nanomater..

[40] Ding M., Cai X., Jiang H.-L. (2019). Improving MOF Stability: Approaches and Applications. Chem. Sci..

[41] Dhakshinamoorthy A., Asiri A.M., García H. (2016). Metal–Organic Framework (MOF) Compounds: Photocatalysts for Redox Reactions and Solar Fuel Production. Angew. Chem. Int. Ed..

[42] Yuan S., Feng L., Wang K., Pang J., Bosch M., Lollar C., Sun Y., Qin J., Yang X., Zhang P. (2018). Stable Metal–Organic Frameworks: Design, Synthesis, and Applications. Adv. Mater..

[43] Molavi H., Hakimian A., Shojaei A., Raeiszadeh M. (2018). Selective Dye Adsorption by Highly Water Stable Metal-Organic Framework: Long Term Stability Analysis in Aqueous Media. Appl. Surf. Sci..

[44] Li Z.-G., Li K., Dong L.-Y., Guo T.-M., Azeem M., Li W., Bu X.-H. (2021). Acoustic Properties of Metal-Organic Frameworks. Research.

[45] Zeleňák V., Saldan I. (2021). Factors Affecting Hydrogen Adsorption in Metal–Organic Frameworks: A Short Review. Nanomaterials.

[46] Li X., Liu H., Jia X., Li G., An T., Gao Y. (2018). Novel Approach for Removing Brominated Flame Retardant from Aquatic Environments Using Cu/Fe-Based Metal-Organic Frameworks: A Case of Hexabromocyclododecane (HBCD). Sci. Total Environ..

[47] Su H., Lv J., Yang L., Feng L., Liu Y., Du Z., Zhang L. (2020). Rapid and Selective Adsorption of a Typical Aromatic Organophosphorus Flame Retardant on MIL-101-Based Metal–Organic Frameworks. RSC Adv..

[48] Zango Z.U., Sambudi N.S., Jumbri K., Abu Bakar N.H.H., Saad B. (2020). Removal of Pyrene from Aqueous Solution Using Fe-Based Metal-Organic Frameworks. IOP Conf. Ser. Earth Environ. Sci..

[49] Mohd Azmi L.H., Williams D., Ladewig B.P. (2020). Can Metal Organic Frameworks Outperform Adsorptive Removal of Harmful Phenolic Compound 2-Chlorophenol by Activated Carbon?. Chem. Eng. Res. Des..

[50] Lv H., Zhao H., Cao T., Qian L., Wang Y., Zhao G. (2015). Efficient Degradation of High Concentration Azo-Dye Wastewater by Heterogeneous Fenton Process with Iron-Based Metal-Organic Framework. J. Mol. Catal. A Chem..

[51] Sun Q., Liu M., Li K., Zuo Y., Han Y., Wang J., Song C., Zhang G., Guo X. (2015). Facile Synthesis of Fe-Containing Metal–Organic Frameworks as Highly Efficient Catalysts for Degradation of Phenol at Neutral PH and Ambient Temperature. CrystEngComm.

[52] Gao C., Chen S., Quan X., Yu H., Zhang Y. (2017). Enhanced Fenton-like Catalysis by Iron-Based Metal Organic Frameworks for Degradation of Organic Pollutants. J. Catal..

[53] Zhou X., Xu D., Chen Y., Hu Y. (2020). Enhanced Degradation of Triclosan in Heterogeneous E-Fenton Process with MOF-Derived Hierarchical Mn/Fe@PC Modified Cathode. Chem. Eng. J..

[54] Zhang M., Wang L., Zeng T., Shang Q., Zhou H., Pan Z., Cheng Q. (2018). Two Pure MOF-Photocatalysts Readily Prepared for the Degradation of Methylene Blue Dye under Visible Light. Dalton Trans..

[55] Ding J., Sun Y.-G., Ma Y.-L. (2021). Highly Stable Mn-Doped Metal–Organic Framework Fenton-Like Catalyst for the Removal of Wastewater Organic Pollutants at All Light Levels. ACS Omega.

[56] Xu J., Shimakoshi H., Hisaeda Y. (2015). Development of Metal-Organic Framework (MOF)-B12 System as New Bio-Inspired Heterogeneous Catalyst. J. Organomet. Chem..

[57] Zango Z.U., Jumbri K., Sambudi N.S., Ramli A., Abu Bakar N.H., Saad B., Rozaini M.N., Isiyaka H.A., Jagaba A.H., Aldaghri O. (2020). A Critical Review on Metal-Organic Frameworks and Their Composites as Advanced Materials for Adsorption and Photocatalytic Degradation of Emerging Organic Pollutants from Wastewater. Polymers.

[58] Zhu D., Hyun S., Pignatello J.J., Lee L.S. (2004). Evidence for Π−π Electron Donor−Acceptor Interactions between π-Donor Aromatic Compounds and π-Acceptor Sites in Soil Organic Matter through PH Effects on Sorption. Environ. Sci. Technol..

[59] Badea S.-L., Mustafa M., Lundstedt S., Tysklind M. (2014). Leachability and Desorption of PCBs from Soil and Their Dependency on pH and Dissolved Organic Matter. Sci. Total Environ..

[60] Wang B., Wang P., Xie L.-H., Lin R.-B., Lv J., Li J.-R., Chen B. (2019). A Stable Zirconium Based Metal-Organic Framework for Specific Recognition of Representative Polychlorinated Dibenzo-p-Dioxin Molecules. Nat. Commun..

[61] Yang L., Liu G., Shen J., Wang M., Yang Q., Zheng M. (2021). Environmental Characteristics and Formations of Polybrominated Dibenzo-p-Dioxins and Dibenzofurans. Environ. Int..

[62] Sharma V.K., Feng M. (2019). Water Depollution Using Metal-Organic Frameworks-Catalyzed Advanced Oxidation Processes: A Review. J. Hazard. Mater..

[63] Zhu G., Wang S., Yu Z., Zhang L., Wang D., Pang B., Sun W. (2019). Application of Fe-MOFs in Advanced Oxidation Processes. Res. Chem. Intermed..

[64] Martínez F., Leo P., Orcajo G., Díaz-García M., Sanchez-Sanchez M., Calleja G. (2018). Sustainable Fe-BTC Catalyst for Efficient Removal of Mehylene Blue by Advanced Fenton Oxidation. Catal. Today.

[65] Bhattacharjee S., Matin M. (2020). Hydroxylation of Phenol Catalyzed by Iron Metal-Organic Framework (Fe-BTC) with Hydrogen Peroxide. J. Mater. Sci. Chem. Eng..

[66] Li X., Guo W., Liu Z., Wang R., Liu H. (2016). Fe-Based MOFs for Efficient Adsorption and Degradation of Acid Orange 7 in Aqueous Solution via Persulfate Activation. Appl. Surf. Sci..

[67] Xue W., Zhou Q., Li F. (2020). The Feasibility of Typical Metal–Organic Framework Derived Fe, Co, N Co-Doped Carbon as a Robust Electrocatalyst for Oxygen Reduction Reaction in Microbial Fuel Cell. Electrochim. Acta.

[68] Wang J., Li B., Wang S., Liu T., Jia B., Liu W., Dong P. (2022). Metal-Organic Framework-Derived Iron Oxide Modified Carbon Cloth as a High-Power Density Microbial Fuel Cell Anode. J. Clean. Prod..

[69] Wang Q., Gao Q., Al-Enizi A.M., Nafady A., Ma S. (2020). Recent Advances in MOF-Based Photocatalysis: Environmental Remediation under Visible Light. Inorg. Chem. Front..

[70] Salazar-Aguilar A.D., Vega G., Casas J.A., Vega-Díaz S.M., Tristan F., Meneses-Rodríguez D., Belmonte M., Quintanilla A. (2020). Direct Hydroxylation of Phenol to Dihydroxybenzenes by H2O2 and Fe-Based Metal-Organic Framework Catalyst at Room Temperature. Catalysts.

[71] Acero J.L., Gunten U. (2000). von Influence of Carbonate on the Ozone/Hydrogen Peroxide Based Advanced Oxidation Process for Drinking Water Treatment. Ozone Sci. Eng..

[72] Huang K., Zhang H. (2022). A Comprehensive Kinetic Model for Phenol Oxidation in Seven Advanced Oxidation Processes and Considering the Effects of Halides and Carbonate. Water Res. X.

[73] Tufail A., Price W.E., Hai F.I. (2020). A Critical Review on Advanced Oxidation Processes for the Removal of Trace Organic Contaminants: A Voyage from Individual to Integrated Processes. Chemosphere.

